# Prognostic impact of antiplatelet therapy in Takotsubo syndrome: a systematic review and meta-analysis of the literature

**DOI:** 10.1007/s10741-021-10099-5

**Published:** 2021-03-29

**Authors:** Francesca Rizzetto, Micaela Lia, Maddalena Widmann, Domenico Tavella, Luisa Zanolla, Michele Pighi, Valeria Ferrero, Flavio Luciano Ribichini

**Affiliations:** Division of Cardiology, Department of Medicine, University of Verona, Verona, Italy

**Keywords:** Aspirin, Takotsubo, Stress cardiomyopathy, Broken heart syndrome, Antiplatelet, Antithrombotic

## Abstract

While the most recent evidence suggests a lack of benefit, antithrombotic therapy is still extensively prescribed in patients with Takotsubo syndrome (TTS). The objective of this study was to determine whether patients with TTS benefit from anti-aggregation, in terms of either short-term or long-term outcomes. A systematic review and meta-analysis was conducted. A comprehensive search of the literature included MEDLINE, Cochrane Library, Clinicaltrials.gov, EU Clinical Trial Register, References, and contact with the authors. Methodological quality assessment and data extraction were systematically performed. The review adhered to the PRISMA framework guidelines. A total of 86 citations were identified, six being eligible for inclusion, for a total of 1997 patients. One of them considered both short-term and long-term outcomes. One reported outcomes during the index event, while the remaining four focused on potential long-term benefits. They were all retrospective cohort studies.

Based on our data, the long-term use of antiplatelet therapy (AT) led to a significantly higher incidence of the composite outcome (OR: 1.54; 95% CI 1.09–2.17; *p* = 0.014) and overall mortality (OR 1.72; 95% CI 1.07–2.77; *p* = 0.027). The analysis did not show a statistically significant difference in TTS recurrences, stroke/TIA, and MI or CAD worsening with AT compared with no anti-aggregation. The AT in this settings did not show any clear benefit in improving the long-term outcomes, and it may be even detrimental and it may be detrimental. These results warrant further future research and the design of adequately powered randomized controlled trials focusing on the impact of aspirin on the outcomes in patients presenting with TTS.

## Introduction

The Takotsubo syndrome is an acute and reversible heart failure syndrome that, at the presentation, could be misdiagnosed as an acute coronary syndrome (ACS) due to its clinical and electrocardiographic characteristics. In this regard, the Mayo Clinic criteria underline the main features of TTS [1], and in the past decades, several authors illustrated the differences between these two clinical conditions [2].

In TTS, there is a close relation between the brain and the circulatory system [3]. The sudden release of catecholamines represents the prominent factor leading to myocardial toxicity. Indeed, catecholamine toxicity is characterized, from an histological standpoint, by the infiltration of mononuclear inflammatory cells, the subsequent fibrotic response, and the characteristic contraction bands [4]. In particular, the interstitial mononuclear inflammatory response seen in the contraction band necrosis with hypercontracted sarcomeres distinguishes the myocyte injury in TTS from the polymorphonuclear inflammation seen in myocardial infarction [5].

Nevertheless, the similarity to ACS often pushes clinicians to prescribe AT, either during hospitalization or lifelong. The awareness could partially explain how stressful events and adrenergic activity induce a blood hyperviscosity state that AT could counteract [6]. Nevertheless, several studies showed AT (either as single or as DAPT) does not reduce major adverse cardiovascular events (MACE). The lack of clear recommendations and gold-standard approaches often causes inadequate therapy administration in this setting.

The main objective of this systematic review with meta-analysis was to assess the effectiveness of aspirin or other antiplatelet agents in reducing either short-term or long-term adverse outcomes in patients with a diagnosis of Takotsubo syndrome.

## Materials and methods

The present systematic review and meta-analysis of the literature adhered to the PRISMA [7] framework.

### Inclusion criteria

Studies were included if they met the following criteria:

- Male and female adult patients, with a diagnosis of TTS according to Mayo Clinic Criteria (2004 criteria up to 2008, then modified Mayo Clinic criteria) [8];
- The exposure measured was the use of either aspirin or P2Y12 inhibitors (eventually as DAPT);
- Reported data for at least two of the following outcomes: overall survival, TTS recurrence, myocardial infarction (MI), stroke;
- Publication date from January 1st, 2007 to August 31th, 2020;
- Either randomized controlled trials, cohort, or case-control studies.

### Exclusion criteria

- Age-specific or ethnicity-specific studies;
- Any literature review, meta-analysis, comment, letter, editorial, and case report for the same population;
- Animal and in vitro studies;
- For the long-term outcome subgroup, a planned median follow-up shorter than 12 months after diagnosis for surviving patients.

Randomized controlled trials (RCTs), meta-analyses, prospective and retrospective cohort studies, and case-control studies were identified from Medline, from Clinicaltrials.gov, from the EU Clinical Trial Register and the Cochrane Central Register of Control Trials. Three independent peer investigators did the same research and came up with the same study selection. No language restriction was deemed necessary.

#### Pubmed

The search strategy string was the following: ("Stress Cardiomyopathy"[All Fields] OR Takotsubo[All Fields] OR Tako-tsubo[All Fields]) AND ("aspirin"[MeSH Terms] OR "aspirin"[All Fields] OR ASA[All Fields] OR Antiplatelet[All Fields] OR "clopidogrel"[MeSH Terms] OR "clopidogrel"[All Fields] OR Antithrombotic[All Fields]). A free terms search was considered more appropriate since MESH terms were found to exclude some important studies.

#### Cochrane central register of controlled trials

The following search strategies were adopted "Takotsubo AND aspirin", "Takotsubo AND antiplatelet", “Takotsubo AND antithrombotic", "aspirin AND stress cardiomyopathy".

#### Clinicaltrials.gov

The following search strategies were adopted “takotsubo AND aspirin”, “takotsubo AND antiplatelet” and “takotsubo AND antithrombotic”.

#### EU clinical trial register

The following search strategy was adopted “Takotsubo”.

All the reference material of the investigated full texts was searched, and where papers cited other potentially relevant references, these were assessed.

Some of the most influential authors about this topic were contacted by email to assess publication and small study bias. They were asked whether they had conducted/knew any further (eventually unpublished) related study and whether they could provide further raw data about included studies.

One reviewer extracted study and patient characteristics, intervention details, and outcome data from included studies with a standardized method. Two other authors checked for accuracy, and disagreements were resolved by consensus.

The following data were extracted: year of the study, Country, study design, sample size population, main characteristics of the population considered, the main outcome, drug type, dosage, follow-up length, outcome variables, statistical analysis, main findings, and conclusions. The potential conflict of interest of the authors was considered. Meta-analysis for short-term outcomes was not deemed appropriate.

### Statistical analysis

Dichotomous outcomes were expressed as odds ratios (ORs) with 95% confidence intervals (CIs). Heterogeneity among included studies was explored qualitatively and quantitatively (using the chi-square test of heterogeneity and *I*^2^ statistic). Data from each study were pooled using a fixed-effects meta-analysis model, due to lack of heterogeneity (for composite outcome heterogeneity *χ*^2^ = 0.21, *d.f.* = 3, *p* = 0.976; for all-cause mortality heterogeneity *χ*^2^ = 1.59, *d.f.* = 2, *p* = 0.451; for TTS recurrence heterogeneity *χ*^2^ = 0.19, *d.f.* = 2, *p* = 0.908; for stroke/TIA heterogeneity *χ*^2^ = 2.03, *d.f.* = 1, *p* = 0.154; for MI/CAD progression heterogeneity *χ*^2^ = 1.50, *d.f.* = 2, *p* = 0.473; for each sub-analysis *I*^2^ 0.0%). A composite outcome was analyzed, and data were then further stratified to explore the effect on each outcome.

Analyses were performed using Stata/IC 14 for Windows (Stata Corp LP, 4905 Lakeway Drive, College Station, TX 77845, USA).

## Results

### Search results

**Fig. 1:**
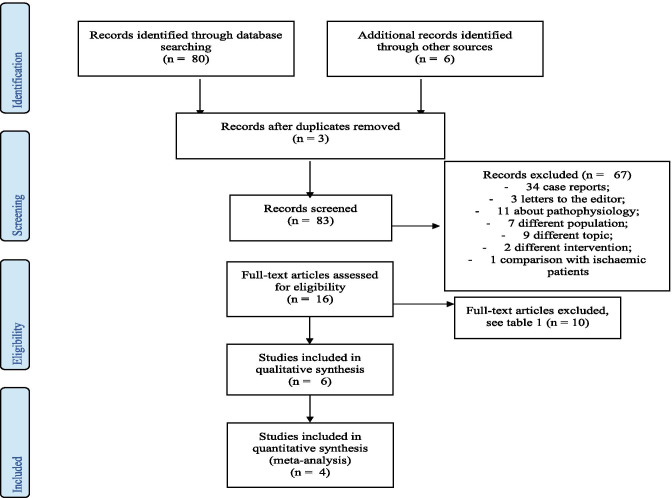
Study flow chart which illustrates the study selection process in accordance with the PRISMA statement. PRISMA: Preferred Reporting Items for Systematic Reviews and Meta-Analyses

Figure 1 shows the flow chart of the study selection process. The search of databases and article references produced 86 references. The removal of duplicates resulted in 83 references. Titles and abstracts of all retrieved papers were then assessed using the pre-specified inclusion criteria. Sixty-eight studies were either case reports/reviews/expert opinions or were considered not related to the main objective of this systematic review, while 15 were selected for retrieval for detailed evaluation: six studies were appropriate for inclusion, while the other nine of them were considered not eligible (Table 1).

**Table 1:** Characteristics of excluded full texts

Parodi et al*.* [9], Cangella et al*.* [10]	No antiplatelet therapy as measured exposure
Luu et al*.* [11], Samardhi et al*.* [12], Opolski et al*.* [13]	The outcome was not reported/stratified depending on antiplatelet therapy prescription
Fazio et al*.* [14]	Ejection fraction recovery and hospitalization length as only reported outcome
Núñez-Gil et al*.* [15]	Laboratory data as outcome
Dias et al*.* [16]	Stroke as only outcome measured; ethnicity subgroup
Santoro et al*.* [17]	Meta-analysis (but its references were investigated with success)

One study was incomplete (not fully published yet), but data of interest were available, and the authors were contacted by email to discuss the availability of further data.

One study focused on in-hospital outcomes (up to 30 days after admission), four described long-term outcomes, and one examined both. Another one gave some information about 30-days outcomes, but these data were incomplete so that any discussion based on them was deemed unreliable [18].

Only four studies were included in both qualitative and quantitative examination (for the long term).

### Description of included studies

All the included studies were observational cohort studies (see Table 2 and 3).

The sample size ranged from 12 to 1533, with 1739 patients for the short-term outcome, and 1791 patients followed up over a long-term period. The mean age of participants was 66.9 years, the great majority of whom were females (consistent with the current epidemiology of Takotsubo syndrome).

Participant’s treatment with AT after receiving a diagnosis of Takotsubo syndrome was compared with no antiaggregation. As extensively shown in Tables 2 and 3, one of the included studies considered short-term outcomes, while four studies considered long-term outcome [18–21] and one reported both [22]. These two outcomes were considered separately.

**Table 2:** Characteristics of included studies for short-term outcome

Author and year	Country	Design	Patients (and cohort by antiplatelet drug use)	Mean age (years)	Male sex (%)	Outcome measured	Length of follow-up	Results
D’Ascenzo et al*.* (2019)	25 Cardio-vascular centres across nine countries (InterTAK Registry)	Multicenter observational Retrospective Cohort Study	1533 (aspirin at discharge: 1031; no antithrombotic therapy: 502)	66.4 ± 13.1	9.8	MACCE	30 days	HR 1.24 (95% CI 0.5–3.04), *p* = 0.64
Dias et al*.* (2015)	USA	Observational Retrospective Cohort Study	206 (87 aspirin alone, 1 clopidogrel alone, 88 DAPT, 30 none)	67.8	13	MACE	Index hospitalization	Single therapy: OR 0.4, 95% CI (0.16–0.9), *P* = 0.04; DAPT: OR 0.34, 95% CI (0.18–0.65)

*MACCE* major adverse cardiovascular and cerebrovascular events,* HR* hazard ratio, *CI* confidence interval, *DAPT* dual antiplatelet therapy, *MACE *major adverse cardiovascular events, *OR* odds ratio

**Table 3:** Characteristic of included studies for long-term outcome

Author and year	Country	Design	Patients (and cohort by antiplatelet drug use)	Mean age (years)	Male sex (%)	Outcome measured	Length of follow-up	Results
D’Ascenzo et al. (2019)	25 cardiovascular centers across nine countries (InterTAK Registry)	Multicenter observational retrospective cohort study	1533 (aspirin at discharge: 1031; none: 502)	66.4 ± 13.1	9.8	MACCE	5 years	HR 1.11 (95% CI 0.78–1.58), *p* = 0.58
Piackova et al. (2018)	Austria	Observational retrospective cohort study	99 (44 aspirin alone, 44 DAPT for 12 months than aspirin, 11 none)	67.8	18.2	All-cause and cardiovascular mortality	5.9 years	CV mortality HR = 0.61; CI = 0.21–1.79, *p* 0.37; all-cause HR = 0.81; CI = 0.45–1.46, *p* = 0.49)
Abanador-Kamper et al. (2016)	Germany	Observational retrospective cohort study	72 (28 on monotherapy; 29 on DAPT; 2 on triple therapy; 4 on OAC + aspirin/clopidogrel; 9 none)	68.8	7	MACE	24 months and 36 months	Low MACE rate compared with existing data. 1 had a stroke on OAC; 1 recurrence in DAPT, 1 MI in monotherapy
Khalighi et al*.* (2016)	USA	Observational retrospective cohort study	12 (6 patients on aspirin, 4 on DAPT; 2 patients with none)	66	0	Death, cardio- shock, SCD, recurrence, re-hospitalization	8.3 ± 3.6 years	No difference
Cacciotti et al. (2012)	Italy	Observational retrospective cohort study	75 (19 were lost during follow-up, so 56, 83.9% on aspirin)	71.9	4	MACE	2.2 years	Both death and recurrence occurred in patients on aspirin

*MACCE* major adverse cardiovascular and cerebrovascular events, *HR* hazard ratio, *CI* confidence interval, *DAPT* dual antiplatelet therapy, *CV* cardiovascular, *OAC* oral anticoagulation, *MACE* major adverse cardiovascular events, *MI* myocardial infarction, *SCD* sudden cardiac death

It should be noted that only the studies on long term outcomes were deemed appropriate for quantitative analysis because an extensive search of the existing literature led to just two studies focusing on short term outcome. The one by Piackova and colleagues was excluded due to the lack of raw data [20].

#### Short-term outcome

The main features of the included studies are summarized in Table 2.

The multicenter observational retrospective cohort study conducted by D’Ascenzo et al. [22] included 1533 consecutive patients from 25 cardiovascular centers with TTS according to the modified Mayo Clinic diagnostic criteria (InterTAK Registry). The cohort was made up mainly of females (1382, 90.2%), and the mean age was 66.4 years, with people ranging from 53.3 to 79.5 years of age. The exposure group included 1031 patients discharged with aspirin, while the other group consisted of the 502 patients discharged without AT.

The outcome (MACE/MACCE) was a composite endpoint of all-cause death, TTS recurrence, stroke, transient ischemic attack (TIA), or MI up to 30 days after the index event.

Unadjusted outcomes showed no difference for all short-term endpoints (unadjusted HR 0.89 [95% CI 0.41–1.95], *p* = 0.78). This result was confirmed by the adjusted analysis based on propensity (HR 1.24 [95% CI 0.5–3.04], *p* = 0.64) and no significant differences were observed for the single components of MACCE (both as unadjusted results and after propensity matching).

In contrast, the retrospective study of Dias et al. [23] suggested a protective role of AT during the index hospitalization. The investigators included 206 patients with TTS admitted between 2003 and 2013. They evaluated whether AT (either aspirin alone or DAPT) was an independent predictor of MACE (defined as in-hospital heart failure, in-hospital death, stroke, or respiratory failure requiring mechanical ventilation) during the index hospitalization. Using a multiple logistic regression model, they demonstrated that single anti-aggregation and DAPT at the time of hospitalization were the only independent predictors of a lower incidence of MACE during index hospitalization [single AT: OR 0.4, 95% CI (0.16–0.9), *p* = 0.04; DAPT: OR 0.34, 95% CI (0.18–0.65), *p* = 0.001].

#### Long-term outcome

As previously mentioned, five out of six studies [18–22] analyzed the association between anti-aggregation after receiving a diagnosis of TTS and long-term outcome.

The cohort considered by D’Ascenzo and colleagues [22] has been described above.

As listed in Table 3, the cohort studied by Piackova included 99 patients with a mean age of 67.8 years, 6.2% of which with a history of MI [20]. It is to notice that the authors decided to exclude patients on oral anticoagulation therapy, and on the one hand, this excludes an important confounding factor. On the other hand, it might represent a selection bias, since due to the risk of apical thrombosis, several TTS patients need anticoagulation. In this paper, multivariable regression analysis was performed to adjust for confounders.

The study by Abanador-Kamper et al. [18] included patients whose average age was 68.8 years, mainly women (93%), but excluded patients with prior MI to clearly distinguish TTS patients. Antithrombotic therapy was prescribed in most patients (96%), making the control group much less represented. Furthermore, some of those not on aspirin received oral anticoagulation (a potential confounder, as reported above).

Finally, even though AT was prescribed for 3 (in one patient) to 12 months (for the great majority), it is compulsory to highlight that therapy was aborted earlier in eleven patients and two other patients received a longer treatment, thus representing a potential bias.

The smallest study was conducted by Khalighi [19] and his group, evaluating only 12 patients (all women), with ten of them receiving either aspirin or DAPT. This study could boast the longest follow-up (8.3 ± 3.6 years).

Finally, Cacciotti and colleagues [21] tried to evaluate the association between aspirin and long-term outcome in a cohort of 75 patients, with 19 of them being lost during the 2.2-year-long follow-up.

Each study individually concluded that aspirin use after diagnosis does not convincingly improve recurrences, mortality, stroke, and other major cardiovascular events. In this regard, the largest study [22] presented unadjusted data showing no differences among the two groups for the composite outcome (unadjusted HR 1.15 [95% CI 0.81–1.68], *p* = 0.41). By further stratifying these results, no difference for single endpoints emerged.

Similarly, according to the PS stratification method, aspirin was not associated with a reduced hazard of MACCE at 5-year follow-up (HR 1.11, 95% CI 0.78–1.58, *p* = 0.58). It is interesting to highlight that results showed no benefit also for the single components of MACCE, including death (HR 1.36, 95% CI 0.79–2.34, *P* = 0.27), TTS recurrence (HR 0.53, 95% CI 0.27–1.03, *P* = 0.06), stroke/TIA (HR 1.52, 95% CI 0.65–3.54, *P* = 0.33), or MI (HR 3.28, 95% CI 0.38–28.28, *P* = 0.28). The only conflicting result was obtained with the propensity score IPTW and PS covariate adjustment methods, which showed some weak association between aspirin use and a risk reduction for TTS recurrence (PS with IPTW, HR 0.47 [95% CI, 0.26–0.83], *p* = 0.01).

Also, Piackova, who evaluated all-cause and cardiovascular mortality, came to the same conclusion, with a hazard ratio of 0.811 (CI = 0.449–1.464, *p* = 0.488) and of 0.611 (CI = 0.209–1.788, *p* = 0.368), respectively [20].

The group of Abanador-Kamper [18] analyzed the occurrence of MACE (defined as recurrence of TTS or myocardial infarction, stroke, or death) during the first 24 months. Three patients of the cohort died from non-cardiovascular causes (neoplasms), three patients experienced a stroke (two of them during the first 30 days, the other after a 12-month follow-up), one suffered from recurrent TTS during the first year, and in one patient MI was documented. All these patients were on AT. The incidence of events was lower than the one reported in other registers so that the authors did not exclude that it could be attributed to the extensive use of AT.

In the study by Khalighi and colleagues [19], only one patient had coronary artery disease (CAD) progression, and this patient was not on aspirin. Finally, in the study by Cacciotti et al. [21], both death and recurrence occurred in patients taking aspirin.

### Quality assessment and risk of bias in included studies

Each study that fulfilled all inclusion criteria was graded for its quality of evidence. Three peer reviewers independently assessed the methodological quality of each study.

Probably, due to the reluctance in avoiding AT, in a syndrome that resembles so much an ACS, none of them was a RCT. Indeed, they were all cohort studies. Furthermore, since Takotsubo is not such a widespread syndrome, non-multicenter studies included a small number of patients, and for the same reasons, they all had a retrospective design.

For this reason, the risk of bias in individual studies was assessed using the Newcastle-Ottawa Quality Assessment Scale (NOS) for observational studies [24, 25] that evaluates the selection of participants, comparability of the groups, and ascertainment of the outcome of interest. The maximum score that could be given was nine points, and only studies with six or more points were considered “high quality” ones. As listed in Table 4, all the included studies reach this threshold, with one of them gaining the maximum score and one study just above the established cut-off.

**Table 4:** Risk of bias based on the Newcastle-Ottawa scale

Study	Selection 1 2 3 4	Comparability 1	Outcome 1 2 3	Total
D’Ascenzo et al*.* (2019)	A*A*A*A*	A*B*	B*A*A*	9
Dias et al*.* (2015)	A*A*A*A*	A*	B*B A*	7
Piackova et al*.* (2018)	B*A*A*A*	A*	B*A*A*	8
Abanador-Kamper et al*.* (2016)	A*A*A*A*	A*	B*A*A*	8
Khalighi et al*.* (2016)	B*A*A*A*		B*A*A*	7
Cacciotti et al*.* (2012)	A*A*A*A*		B*A*C	6

Selection: 1 Representativeness of the exposed subjects (a-truly representative of the average TTS population in the community*, b-somewhat representative of the average TTS population in the community*, c-selected group of users, e.g., nurses, volunteers, d-no description of the derivation of the cohort); 2 selection of the non-exposed cohort (a-drawn from the same community as the exposed cohort*, b-drawn from a different source, c-no description of the derivation of the non exposed cohort); 3 ascertainment of exposure (a-secure record (eg surgical records)*, b-structured interview*, c-written self report, d-no description); 4 demonstration that outcome of interest was not present at start of study (a-yes*, b-no)

Comparability: 1 Comparability of cohorts based on the design or analysis, with a maximum of two asterisks (a-study controls for age, b) study controls for any additional factor)

Outcome: 1 Assessment of outcome (a-independent blind assessment*, b-record linkage*, c-self report, d-no description), 2 was follow-up long enough for outcomes to occur (a-yes*, b-no), 3 adequacy of follow-up of cohorts (a-complete follow-up*, b-subjects lost at follow-up unlikely to introduce bias*, c-follow-up rate < 60%, d-no statement)

Nevertheless, the majority were formally underpowered for the main outcomes, and the population was skewed towards the AT group.

### Meta-analysis

**Fig. 2:**
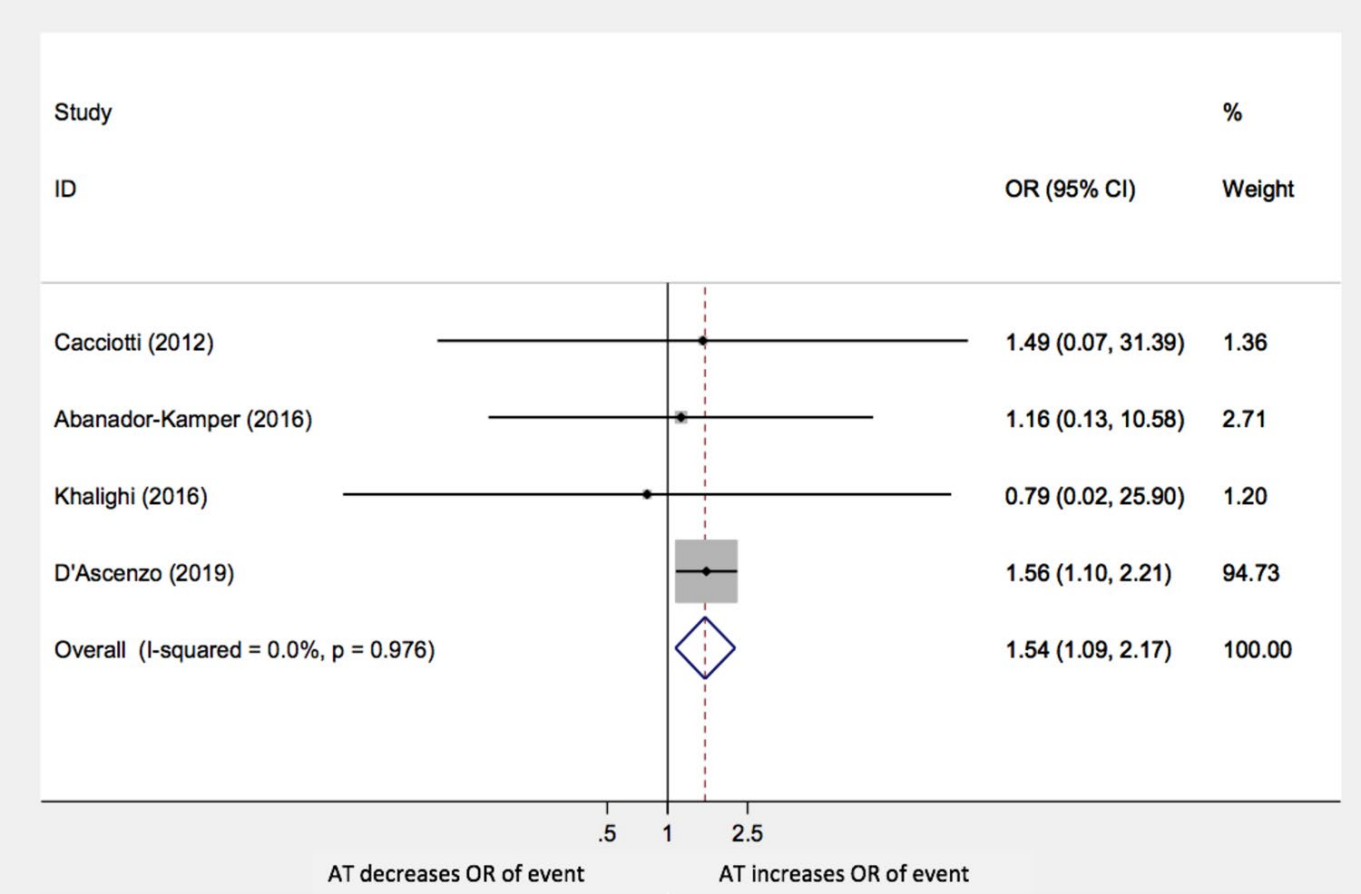
Composite outcome AT: antiplatelet therapy; OR: odds ratio; CI: confidence interval

Our study showed that the administration of any AT following TTS had a negative impact on the long-term composite outcomes (OR: 1.54; 95% CI 1.09–2.17; *p* = 0.014) as shown in Fig. 2.

**Fig. 3:**
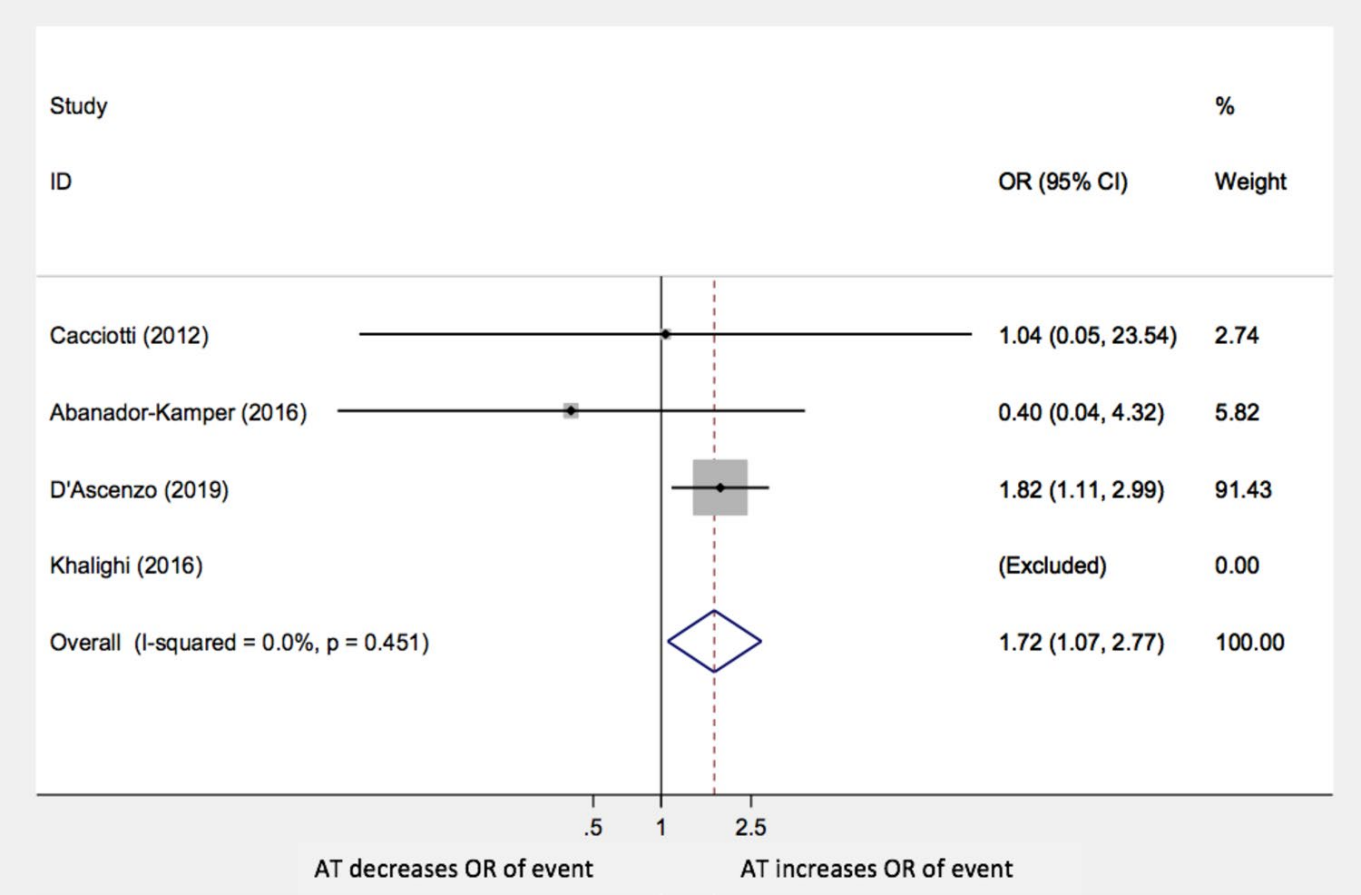
All-cause mortality AT: antiplatelet therapy; OR: odds ratio; CI: confidence interval

The same conclusion can be drawn for all-cause mortality alone (OR 1.72; 95% CI 1.07–2.77; *p* = 0.027), as Fig. 3 shows. The study by Khalighi et al. was not included in this sub-analysis due to the lack of events.

**Fig. 4:**
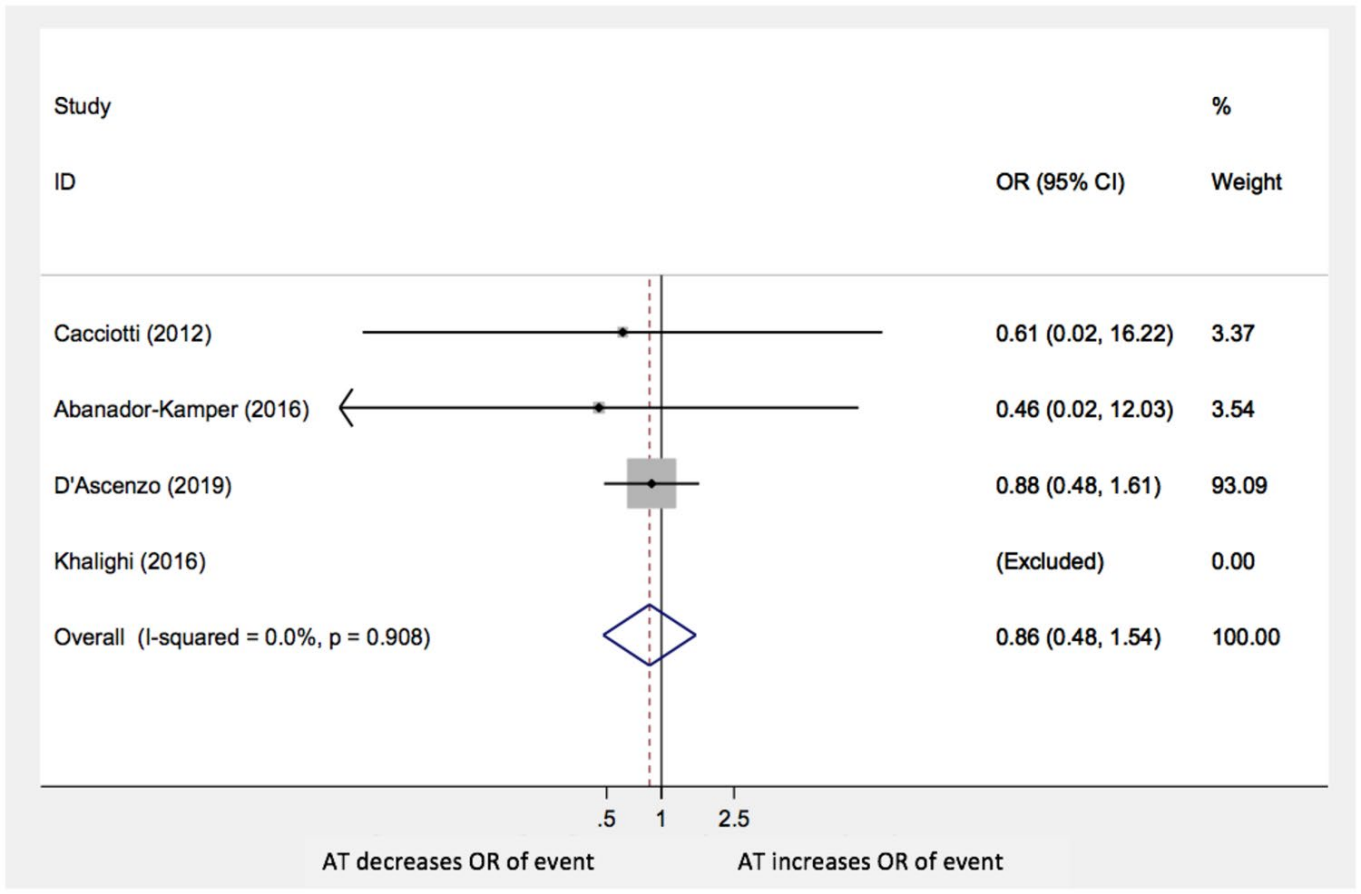
Recurrence of events AT: antiplatelet therapy; OR: odds ratio; CI: confidence interval

**Fig. 5:**
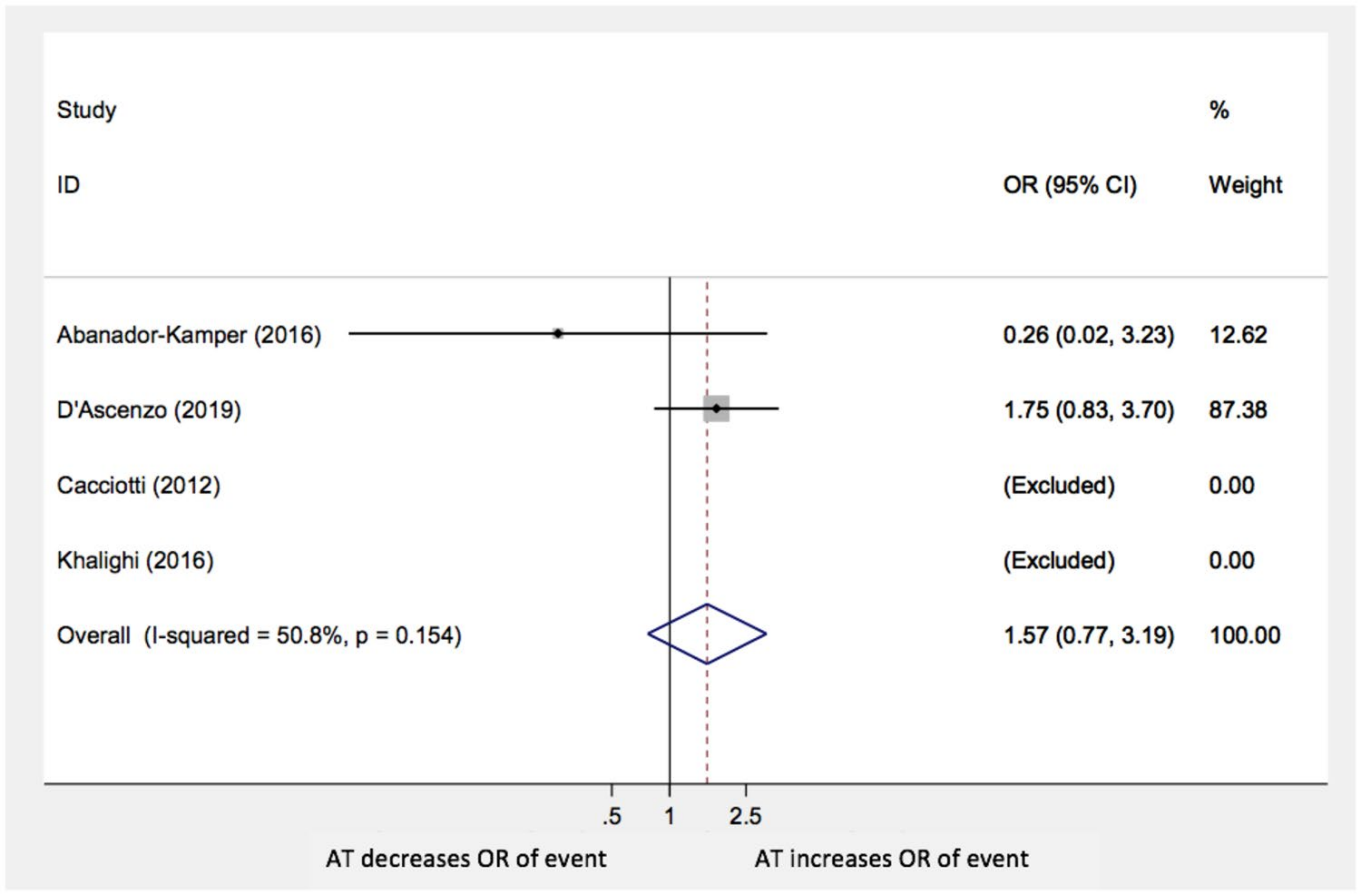
Stroke or TIA AT: antiplatelet therapy; OR: odds ratio; CI: confidence interval

**Fig. 6:**
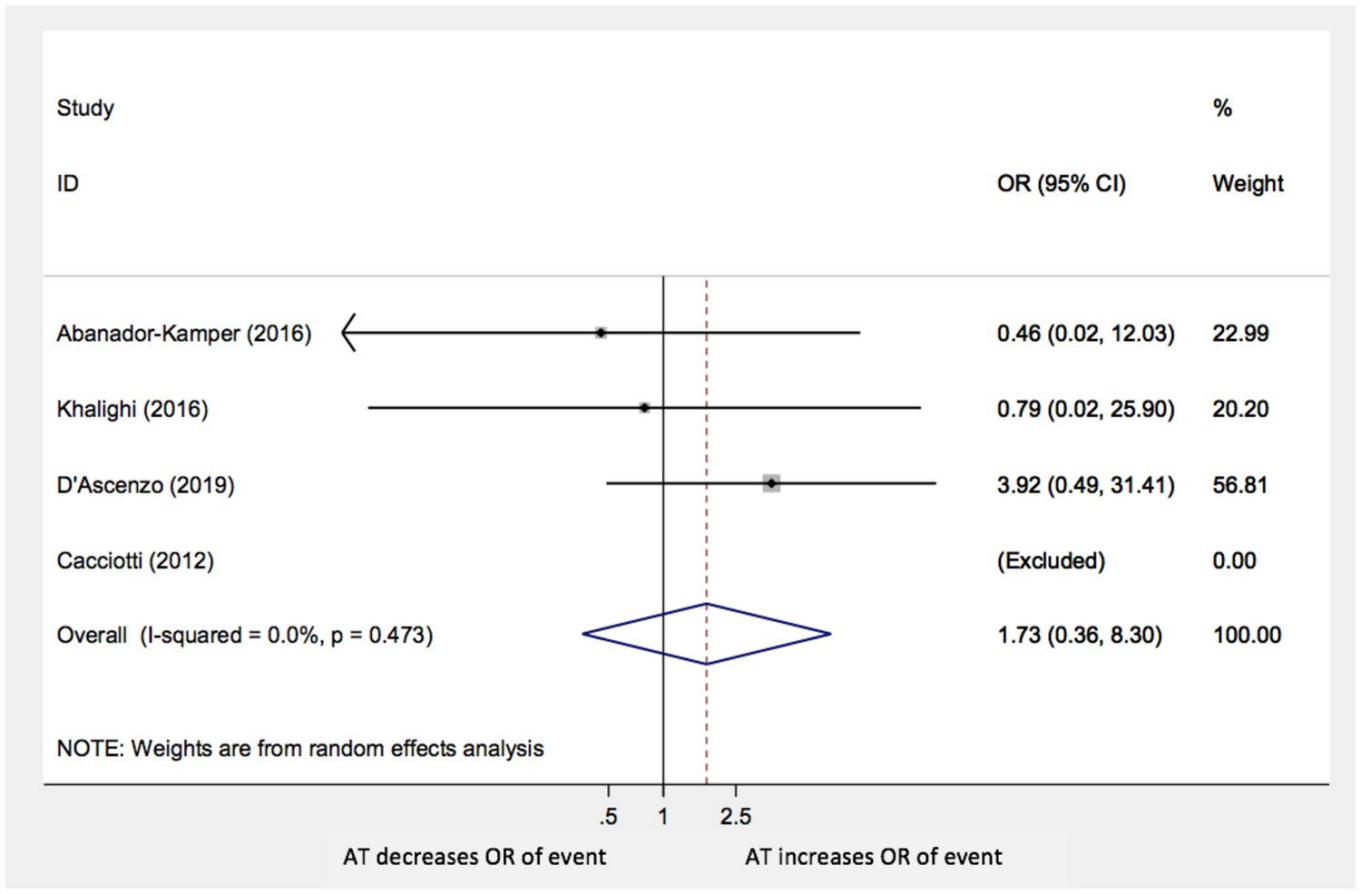
MI or CAD progression AT: antiplatelet therapy; OR: odds ratio; CI: confidence interval

Moreover, the analysis did not show statistically significant differences in TTS recurrences (OR: 0.86; 95% CI: 0.48–1.54; *p* = 0.6), stroke/TIA (OR: 1.57; 95% CI 0.77–3.19; *p* = 0.22), and MI or CAD worsening (OR 1.73; 95% CI 0.36–8.3; *p* = 0.5) with AT compared with no anti-aggregation (Figs. 4, 5, and 6, respectively**)**. The paper by Cacciotti and his group was excluded in both these last two sub-analyses because no events were reported.

## Discussion

Although no RCTs were found, the present meta-analysis collected all the existing studies focusing on the topic, through a systematic review of the literature.

The analysis of the short-term outcome was possible only in two studies which showed contradictory results. Dias et al. [23] focused on the need for aspirin administration during hospitalization, showing a potential benefit, most likely related to the well-known state of blood hyperviscosity [6] characterizing the acute phase of TTS. However, this phenomenon was not confirmed by D’Ascenzo et al. [22], who analyzed a larger cohort of patients for a much more extended period after the index event (30 days). Therefore, the available data to date do not provide any substantiated and univocal message about administering AT during hospitalization.

Concerning the long-term outcome, the present meta-analysis did not significantly show a benefit of single or double AT compared with no antiplatelet therapy. It indeed even provided evidence of an independent negative effect of aspirin, P2Y12 inhibitors or DAPT on long-term overall prognosis and all-cause mortality. The forest-plot shows that D’Ascenzo’s study overbalances the results due to its much higher numerosity.

Although the large sample size, the results of the study conducted by D’Ascenzo and his group did not reach statistical significance. This fact shows that our paper, obtained by cumulative analysis of all existing studies on this issue, leads for the first time to some original statistically significant results. We suggest that the statistically significant result of the present study could give practical guidance for everyday practice, thus filling the gap left by existing evidence and current guidelines. Indeed, the last guidelines (2020) [26] on Acute Coronary Syndrome management in patients presenting without persistent ST-segment elevation do not provide any recommendation on antiplatelet therapy in TTS. Despite this, the same guidelines do not include TTS among the non-obstructive myocardial infarction (MINOCA) due to the lack of an ischemic pattern, making AT therapy inappropriate even from a pathophysiological point of view. This lack of clear recommendation often leads to prescriptions that are not supported by evidence, with two out of three cardiologists who do not adhere to any guidelines and with many patients being dismissed on antiplatelet therapy [27, 28].

The present analysis did not demonstrate any significant difference in TTS recurrence, cerebrovascular events, MI, or CAD progression. Sub-analysis concerning bleeding events could not be performed due to lack of data in most of the included studies.

Finally, the study by Piackova et al., which was not included in quantitative analysis, showed no statistically significant correlation between aspirin/P2Y12-i/DAPT and long-term MACEs.

Based on our data, a widespread prescription of aspirin in this specific setting and in the absence of other strong cardiovascular indications may be not supported, even if we recognize the quality of the evidence itself is limited. Indeed, although based on a superficial analysis, aspirin might seem a harmless drug, it is necessary to bear in mind its adverse effects such as gastrointestinal affections, minor and major bleedings, or allergies. The prescription of antiplatelet therapy for primary prevention is indeed subject to debate. Recent studies, such as the ASPREE [29], showed an increased bleeding risk in patients on aspirin, in the absence of other clinical benefits. The CHARISMA [30] demonstrated an even more detrimental effect of DAPT when compared with single antiplatelet therapy for primary prevention, showing a significant increase of death from any cause and cardiovascular mortality in patients treated with both aspirin and clopidogrel versus aspirin alone. The potential harm of the antiplatelet therapy seems to be related to an increased risk of major (such as gastrointestinal bleeding, cerebrovascular bleeding) and minor (gums or ocular bleeding, epistaxis) bleedings. This phenomenon impacts particularly on frail older people, who are often affected by either neurological disorders or rheumatologic diseases, and who are thus more prone to accidental falls.

In this regard, the lack of data about bleeding risk represents one of the greatest limitations of the included studies but based on the evidence mentioned above, it is reasonable to expect that such sub-analysis would further strengthen the concept that antiplatelet therapy in this setting might be not only useless but even detrimental.

### Study limitations

The main limitation of this meta-analysis is that no RCT has ever been conducted on this topic so that patients with other cardiovascular diseases were more prone to receive AT. Indeed, previous CAD, diabetes mellitus, peripheral artery diseases, and other co-morbidities which may have indicated antiplatelet therapy were not accounted for, with the only exception of Piackova et al*.*, who excluded patients with prior myocardial infarction. Anyway, it should be highlighted that given the low prevalence of TTS, observational studies do currently represent the only available evidence in this setting. Secondly, no subgroup analysis was made depending on the AT type, due to the lack of these information in some of the studies. Thirdly, in most studies, groups were not equally represented, since the vast majority of patients received a prescription of AT, but this further underlines the need to understand its real indications better. According to the Newcastle-Ottawa Scale, all our studies should be considered high-quality ones. Besides, the heterogeneity was low for all the included studies. Nevertheless, may be still some systematic biases, which the 95% confidence interval cannot account for.

Concerning other methodological limitations, all the studies included in the present analysis, but one was underpowered. However, the strength of the study lies in the systematic definition, collection, and analysis of data, as well as in the analysis of MACEs and not of surrogate markers such as ejection fraction recovery.

## Conclusions

This literature review was the first and only comprehensive systematic review with a meta-analysis that evaluated the relationship between AT use after TTS diagnosis and both short-term and long-term outcomes.

This review revealed that no existing evidence supports lifelong AT in patients with TTS. In particular, anti-aggregation is of no use, but for the first time, we also found a statistically significant result suggesting a detrimental role of AT for composite MACEs and overall mortality.

The conflicting results about short-term outcomes suggest that this is an area worthy of further research. No RCT was ever conducted, and our analysis represents the state-of-the-art and the highest level of evidence the entire literature provides. We believe that it could help the clinician in treating Takotsubo patients (corroborating the fact that aspirin may be of harm and that TTS cannot be equated to ischemic cardiopathy) and that it encourages the design of RCTs.

### Implications for research and practice

The need for further investigations on this issue and proceeding with new and more powered cohort studies and RCTs would be advisable, to further assess this item and reassure clinicians about the safety of avoiding aspirin prescription in TTS patients, hopefully addressing more practical concerns also about side effects.

## Abbreviations

TTS: Takotsubo syndrome
ACS: Acute coronary syndrome
MI: Myocardial infarction
PRISMA: Preferred Reporting Items for Systematic Reviews and Meta-Analyses
AT: Antiplatelet therapy
DAPT: Double antiplatelet therapy
MACE: Major adverse cardiovascular events
MACCE: Major adverse cardiovascular and cerebrovascular events
RCTs: Randomized controlled trials
CAD: Coronary artery disease
TIA: Transient ischemic attack
NOS: Newcastle-Ottawa quality assessment Scale
